# A systematic review of measures of emotion regulation in forensic settings

**DOI:** 10.3389/fpsyg.2025.1696832

**Published:** 2026-01-06

**Authors:** Adam Meddeb, Carlo Garofalo, Steven M. Gillespie, Josanne D. M. van Dongen, Malin Hildebrand Karlén, Märta Wallinius

**Affiliations:** 1 Department of Clinical Sciences Lund, Psychiatry, Lund University, Lund, Sweden; 2 Center of Ethics, Law and Mental Health, Institute of Neuroscience and Physiology, The Sahlgrenska Academy, University of Gothenburg, Gothenburg, Sweden; 3 Department of Research, Regional Forensic Psychiatric Clinic, Växjö, Sweden; 4 Department of Philosophy, Social Sciences and Education, University of Perugia, Perugia, Italy; 5 Department of Primary Care and Mental Health, University of Liverpool, Liverpool, United Kingdom; 6 Department of Psychology, Education and Child Studies, Erasmus University Rotterdam, Rotterdam, Netherlands; 7 Department of Psychology, University of Gothenburg, Gothenburg, Sweden

**Keywords:** emotion regulation, forensic, measurement, psychiatry, reliability, validity

## Abstract

**Background:**

The study of emotion regulation (ER) has gained traction in forensic psychological and psychiatric research as a correlate of antisocial and aggressive behavior as well as for its relevance to psychopathology. However, conceptual and definitional ambiguity persists.

**Methods:**

This pre-registered systematic review aimed to investigate how ER is conceptualized and measured in forensic populations, and to synthesize available evidence on the reliability and validity of ER measurement instruments. A total of 59 studies met the eligibility criteria and were included in the review.

**Results:**

ER was primarily assessed using self-report questionnaires (93% of studies), with only four studies employing biophysiological indices of ER. Seven distinct measurement models were identified. Most studies (80%) relied on one of two broad conceptual approaches: ER conceptualized as a set of interrelated abilities, most commonly assessed using the Difficulties in Emotion Regulation Scale, or ER conceptualized as a set of strategies used to regulate emotional responses, most commonly assessed using the Emotion Regulation Questionnaire. Reliability estimates were reported in 64% of studies, with the majority of estimates exceeding conventional cut-offs for adequate internal consistency. Evidence for construct validity was generally conclusive or mixed across studies.

**Conclusions:**

ER research in forensic settings is characterized by conceptual heterogeneity and a strong reliance on self-report measures. The conceptual heterogeneity underscores the need for authors to clearly outline how ER is conceptualized and theoretically defined. Although reliability estimates were generally adequate when available, reliability was not consistently reported across studies.

**Systematic review registration:**

https://www.crd.york.ac.uk/PROSPERO/view/CRD42023495577.

## 1 Introduction

Emotion regulation (ER) may parsimoniously be defined as the process by which emotions are identified, managed and controlled (Gross, 1998; Thompson, 1994). One influential framework conceptualizes ER as a set of specific abilities, namely: emotional awareness, emotional acceptance, emotional clarity, the ability to maintain goal-directed behavior under emotional distress, the ability to use strategies to change emotions and the ability to refrain from impulsive behavior under emotional distress (Gratz and Roemer, 2004). These varying abilities can be measured using the Difficulties in Emotion Regulation Scale (DERS; Gratz and Roemer, 2004), with impairments in one or more of these abilities being characteristic of emotion dysregulation. Other frameworks of ER include the process model of emotion regulation (Gross, 1998; Sheppes et al., 2015), within which ER is defined as the goal-directed process of changing emotions as they develop over time. Within this model, emotions are generated through a sequence of steps with each step representing a point where an individual can intervene (i.e., regulate) to change the course of the emotion trajectory. A range of strategies may be employed at different steps of the emotion generation process, and Gross (1998) classifies these strategies into two broad categories, namely antecedent-focused and response-focused strategies. Antecedent-focused strategies are attempts to regulate emotions that take place before the emotions have fully manifested (i.e., early in the emotion generative lifecycle). A common example of such a strategy is cognitive reappraisal, which entails an individual’s effort to reconstrue the emotion eliciting situation in less emotionally arousing terms, using reinterpretation or psychological distancing. Response-focused strategies, on the other hand, are strategies that an individual employs once the emotion(s) have manifested (i.e., late in the emotion generative cycle). Suppression may exemplify such a strategy as it entails the inhibition of the outward expression of an emotion once the emotion is fully experienced. Although other approaches and definitions have been put forward (see Bloch et al., 2010 for a review), the DERS framework and the process model of ER have been highly influential within ER research.

The field of ER has seen a large increase in research attention over the past few decades. Impairments in ER have been conceptualized as a transdiagnostic factor implicated in the etiology of different psychopathologies (Aldao et al., 2016), and attempts to promote adaptive ER functioning have subsequently been incorporated into different treatment approaches (Gratz et al., 2015; Moltrecht et al., 2021), with a growing body of evidence suggesting that ER is amenable to treatment (Cohen and Ochsner, 2018; Daros et al., 2021). Although the bulk of applied research on ER has examined associations with specific forms of psychopathology (e.g., borderline personality disorder), a growing body of work now exists that suggest that ER is of clinical importance in understanding aggression and antisocial behavior (Garofalo et al., 2022; Roberton et al., 2014; Roberton et al., 2015). Robust links between dysfunctional ER and aggression have been reported across diverse samples, including offenders (Garofalo et al., 2019; Garofalo et al., 2022; Roberton et al., 2014), forensic psychiatric patients (Meddeb et al., 2023) and community samples (Garofalo et al., 2019; Garofalo et al., 2022; Scott et al., 2015). These associations hold even when controlling for important covariates such as anger and antisocial cognition (Roberton et al., 2014; Roberton et al., 2015) and have been further corroborated in a recent systematic review that has found that aggression and maladaptive ER are consistently associated across samples and operationalizations (Navas-Casado et al., 2023). Meta-analytical studies are now accumulating that report on the association between ER and various forms of externalizing behaviors such as intimate partner violence (Maloney et al., 2023), substance abuse (Stellern et al., 2023) and psychopathy (Velotti et al., 2024). Empirical evidence now supports ER as an important dynamic risk factor in sexual offending (Seto et al., 2023), and ER skills are increasingly a focus in treatment programs for sexual offenders (e.g., Olver and Riemer, 2021). These studies highlight that ER may be considered an important treatment target in forensic populations. One conceptual distinction of ER that has been highly influential within forensic psychological research is that between under- and over-regulation of emotions among offenders (Megargee, 1966; Roberton et al., 2014; Davey et al., 2005). Under-regulation refers to failing to contain the emotional experience—acting out in dysfunctional and aggressive behavior. Over-regulation entails continuously inhibiting or suppressing unpleasant emotions, building up internal tension and thus reducing cognitive capacities to resolve emotional issues, which increases the likelihood of aggressive behavior. Other research has been centered around the effectiveness of individual ER strategies (Roberton et al., 2014). For instance, within the original process model of ER, suppression was assumed to be an inherently maladaptive strategy, whereas cognitive reappraisal was characterized as an inherently adaptive strategy. Although much empirical research has assumed the inherent adaptive and maladaptive nature of specific ER strategies, recent research has emphasized the contextual nature of ER (Aldao et al., 2015; Doré et al., 2016). In other words, the effectiveness of ER depends on situational and personal factors and not solely on the type or use of a specific ER strategy. However, the contextual adaptiveness of specific ER strategies in forensic populations has, to our knowledge, not been extensively investigated.

While the evidence-base for ER—variously defined—as a clinical construct of interest in forensic psychology has grown, debate has persisted as to what constitutes ER (Gross and Feldman Barrett, 2011; Gross and Jazaieri, 2014; Tull and Aldao, 2015). In other words, while studies agree on the importance of ER, there is a lack of a unifying conceptualization and operationalization of ER. While the DERS framework approaches ER as a set of diverse interrelated abilities, the process model situates ER within the emotion generating process focusing more on specific attempts (i.e., strategies) to change the emotion as it evolves over time.

Perhaps due to the lack of consensus on definitions of ER, there is currently a wealth of different ER measurements that operationalize the construct in different ways. A recent review of ER measures within youth and adolescent samples identified a total of 87 self- or informant reported measures of ER (Mazefsky et al., 2021). Notably, in this study, less than half of these measures were deemed to be psychometrically and empirically sound based on indices of reliability and validity. This is worrisome, as access to valid and reliable assessments is paramount in understanding ER and its application in forensic psychiatry. On a more general note, the proliferation of psychological measurements has been criticized on the grounds that it may introduce undue heterogeneity in research findings, hampering the accumulation and synthesis of scientific research (Elson et al., 2023). Although previous work has evaluated how ER is conceptualized and measured, this research has primarily been conducted with respect to child and adolescent populations (e.g., Adrian et al., 2011; Mazefsky et al., 2021). No previous research, to our knowledge, exists that has reviewed how ER is conceptualized and measured within forensic psychological research, let alone the reliability and validity of ER assessment in such settings.

## 2 Current study

Over the past two decades ER has increasingly been studied in relation to aggressive and antisocial behavior. ER now figures in general as well as specific theories of crime and is commonly incorporated into treatment programs that aim to reduce the risk of recidivism. However, there is a lack of conceptual clarity as to what ER is and consequently a large heterogeneity in how it is measured across forensic psychological research. This systematic review aims to clarify how ER is measured and conceptualized across forensic settings and evaluate evidence of validity and reliability for those measures that are currently in use. The following research questions were defined to guide this study:

- How is ER measured and conceptualized across forensic settings?
- What evidence for reliability and validity exist for ER measures within forensic settings?

## 3 Materials and methods

This review was conducted in accordance with the Preferred Reporting Items for Systematic Reviews and Meta-Analyses (PRISMA; Moher et al., 2009; Page et al., 2021) guidelines. Given that our research question concerned *measurement instruments* rather than intervention effects, the PICO framework was not compatible with the overarching aims and design of this study. Consistent with prior methodological reviews and COSMIN guidance (Mokkink et al., 2018), we therefore provide a detailed description of the *target population* and *construct of interest* in subsection 3.3, and note that the “intervention” and “comparison” elements are not applicable in this review.

### 3.1 Systematic review protocol

The review, including the search strategy and inclusion and exclusion criteria, was registered on PROSPERO, on 31 December 2023 [CRD42023495577]. Two deviations from the protocol were made during the study. Firstly, quality assessment through the Quality Assessment Tool for Quantitative Studies (QATQ; Thomas et al., 2004) was not judged appropriate for the studies of cross-sectional design. Therefore, we complemented the quality assessment with the Appraisal tool for Quantitative Studies (AXIS; Downes et al., 2016) specifically for those studies of cross-sectional design. Secondly, rather than reporting on correlations between ER measures within studies that employed two or more measurements of ER, we choose to gauge the validity of ER through looking at how measures of ER—variously operationalized—correlated with other constructs of interest to the field. This procedure aims at providing an integrative overview of the construct validity of ER.

### 3.2 Search strategy and data sources

Relevant search terms were determined by the entire research team and in consultation with an information specialist at Lund University, Sweden. Two search blocks were created to identify studies that (1) administered an ER measure (2) in a forensic setting. The first search block employed a broad set of search terms to capture ER more broadly. The second block of search terms aimed at restricting our search to studies where ER was measured in a forensic setting. The following search terms were used: Emotion* regulation OR affect* regulation OR emotion* dysregulation OR dysregulation of emotion OR regulation of emotion OR adaptive emotion regulation OR mood regulation OR emotion repair OR emotion control OR cognitive reappraisal OR distraction OR emotional awareness OR emotional avoidance OR emotional acceptance OR emotion* intelligence AND Forensic OR prison OR forensic psychiatr* OR offender OR probation OR criminal OR antisocial. The search was performed on 2024-02-23 with no date restrictions. The following databases were searched: PubMed, Scopus, PsycINFO, Web of Science (WoS) and EMBASE. A follow-up search employing the same search strategy was conducted 2024-11-18 to update the search with the most recent literature.

### 3.3 Eligibility criteria

All quantitative, peer reviewed studies, published in English and with no restrictions on date were included in the review. Case reports, editorials and review articles were excluded. Due to the lack of a universal definition of ER, we chose to operationalize ER in line with the multi-dimensional framework (Gratz and Roemer, 2004). More specifically, studies were included if they employed an ER measure that tapped one or more of the following dimensions:

1. Awareness of/attention to emotions
2. Understanding of emotions
3. Acceptance of emotions
4. Ability to refrain from impulsive behavior when under emotional pressure
5. Ability to pursue goal-directed behavior under emotional distress
6. Ability to use ER strategies to serve the situation/goals

Measures were included if they assessed the use of specific strategies to regulate emotions. For example, studies that assess cognitive reappraisal or emotion suppression through the Emotion Regulation Questionnaire (ERQ; Gross and John, 2003) would be included. Importantly, measures that assessed some of the above dimensions as well as other (irrelevant) constructs were not included (e.g., broadband measures of psychological functioning) because (1) we wanted to balance feasibility with comprehensiveness, (2) identifying keywords to capture all such measures proved conceptually challenging, and (3) our aim was to identify measures of ER in particular that could be recommended for use in forensic settings. Constructs that might overlap with ER but were not considered eligible for inclusion in this review include alexithymia, emotional intelligence, and mindfulness. For example, alexithymia, as measured through the Toronto Alexithymia Scale (TAS-20; Bagby et al., 1994), was not considered a measure of ER since TAS-20 also measures the degree of externally oriented thinking. Similarly, emotional intelligence, as measured through the Emotional Quotient Inventory (EQ-I; Bar-On, 1997) or the performance-based Mayer-Salovey-Caruso Emotional Intelligence Test (MSCEIT; Mayer et al., 2002), were not considered measures of ER as these measures tap cognitive abilities such as empathy and using emotions to facilitate thought. No restrictions in terms of measurement method were applied to allow for a variety of measurement approaches for the assessment of ER, including neurobiological/physiological measures, self-report measures and performance-based measures. Our second inclusion criterion related to the population being sampled—the target population. Studies were included if they employed a measure of ER as detailed above in a forensic setting such as a prison or a forensic psychiatric hospital. Forensic settings were defined as all such settings that aim at treating or incarcerating individuals who are at risk of being convicted, or who have been convicted, of a crime. Forensic settings eligible for inclusion included both inpatient and outpatient settings. Lastly, only adult samples defined as having a mean age > 18 years were included in the review.

### 3.4 Study selection procedure

Study abstracts and titles were screened by author AM. At the full-text screening, author AM and MW screened studies independently and met for discussion and dialogue for resolving conflicts. All data extraction was performed by author AM, in dialogue with author MW.

### 3.5 Quality assessment

Quality assessments were performed using the AXIS for studies of cross-sectional design, and using the QATQ for studies with an experimental or longitudinal design. The QATQ rates studies within six domains, namely: selection bias, study design, confounders, blinding, data collection methods, withdrawals and drop-outs. A global score is provided as a function of the frequency of domains rated as weak with the occurrence of two or more weak components providing an overall weak rating, with one weak rating providing an overall moderate rating and with the absence of any weak components providing an overall strong rating. The AXIS critically appraises studies of cross-sectional design through 20 questions. Each question is answered with a “yes” or “no.” Although the AXIS does not yield a global rating of study quality, we coded ratings dichotomously (1 = question fulfilled, 0 = question not fulfilled) giving a scoring interval of 0–20. Studies that were scored >18 (90%) were coded as strong, >14 (70%) as moderate and <14 (<70%) as weak. Global ratings are provided in the results section and the interested reader may access component ratings in the raw data sheet.

### 3.6 Data extraction and coding procedures

A novel data extraction protocol was developed to help structure the data extraction procedure. Author AM extracted the following data from all the identified studies: (1) study characteristics, (2) sample characteristics, (3) measurement characteristics, and (4) study results. In study characteristics, we coded main author, journal, publication year, country and study design. Sample characteristics included various aspects of the study sample such as sample size, age and gender composition. The forensic setting that participants were sampled from was also coded to indicate a prison setting, a forensic psychiatric setting, or a combination of both, and whether participants were inpatients or outpatients, or a mixture of both. Measurement characteristics included ER measure, estimates of reliability and internal consistency, measures of central tendency and standard deviations. In line with previous literature (Tull and Aldao, 2015) we coded if ER was conceptualized through a strategy-based framework (i.e., assessing one or several specific strategies such as suppression, cognitive reappraisal and rumination) or an ability-based framework (i.e., assessing broader tendencies in how individuals understand, value and respond to their emotions). If individual studies expanded on how ER was defined or conceptualized, we recorded such notes for further analysis. In studies that employed mixed samples, including both offenders and non-offenders, information was only extracted from the forensic sample. When repeated measures of ER were administered, we only extracted information from the first assessment. In studies that reported measurement characteristics for more than one forensic sample, pooled standard deviations and mean scores were calculated for the combined forensic population.

Lastly, in study results we coded the other variables measured alongside ER. In order to examine validity, we assessed studies on whether zero-order correlations between ER and other measured constructs were in line with theoretical expectations. We expected that ER would be empirically related to various measures of psychopathology considering the burgeoning evidence of ER as a transdiagnostic feature across both externalizing and internalizing pathology (Aldao et al., 2016). On the basis of correlation matrices, we coded whether studies provided support for validity (i.e., all empirical relationships were in line with theoretical expectations), mixed support of validity (i.e., some empirical relationships were in line with theoretical expectations but not all) and no support of validity (i.e., no empirical relationships were in line with theoretical expectations). In order to summarize findings across studies, we aggregated relationships across studies such that all studies that examined any form of association between ER and aggression were considered to investigate the same conceptual relationship (i.e., the relationship between aggression and ER) independent of how aggression or ER were operationalized in each specific study. Through this methodology, we aimed at providing an integrative picture of the construct validity of ER across the spectrum of constructs in the nomological network of ER that were investigated in forensic samples.

### 3.7 Data analysis

All data was handled in the R programming language (version 4.4.0; R Core Team, 2023) and tables and figures were created through the ggplot2 package (Wickham, 2016) and the PRISMA2020 Shiny application (Haddaway et al., 2022). Raw data and all code used in this article are publicly and freely available at.^1^

## 4 Results

### 4.1 Study selection

**Figure 1:**
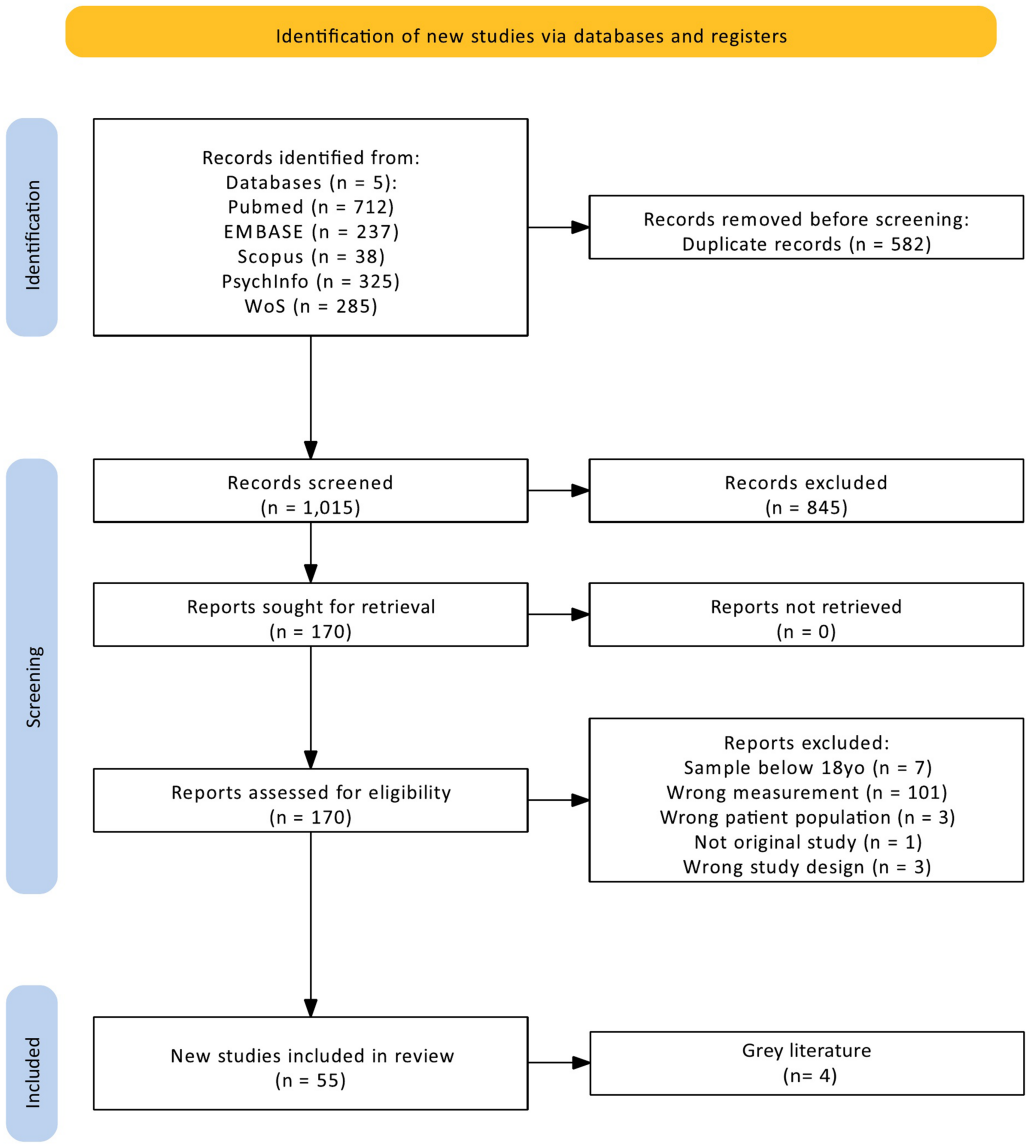
PRISMA flowchart.

A total of 1,524 studies were identified across the five databases, with 967 studies remaining for abstract- and title screening after duplicates were removed. Following abstract- and title screening, 159 studies eligible for full-text screening remained. During full-text screening, disagreements occurred across 28 records (18%), which were resolved through discussions between author AM and MW and mainly centered around the operationalization of ER. Specifically, discussions revolved around studies that operationalized ER through the Cognitive Emotion Regulation Questionnaire (CERQ; Garnefski and Kraaij, 2007), the Emotion Control Questionnaire (ECQ; Roger and Nesshoever, 1987) and the Brief-Coping Orientation to Problems Experienced Questionnaire (B-COPE; Carver, 1997). Items for each questionnaire were reviewed and we choose to retain the CERQ as this measure taps intraindividual strategies individuals use to change their emotions and exclude the ECQ and the B-COPE as we judged these to measure coping more widely. Studies that sampled individuals with an age range including minors were excluded from further analysis (*n* = 7), as well as studies that used a qualitative design (*n* = 3) or were conference abstracts (*n* = 1). A total of 48 studies were included in the review after the full-text screening with an additional four studies identified through the reference section of the identified records. The follow-up search resulted in a further 73 studies identified, with seven retained in this review. In total, 59 eligible studies were included in the review. A PRISMA flowchart is provided in Figure 1.

### 4.2 Characteristics of included studies

**Figure 2:**
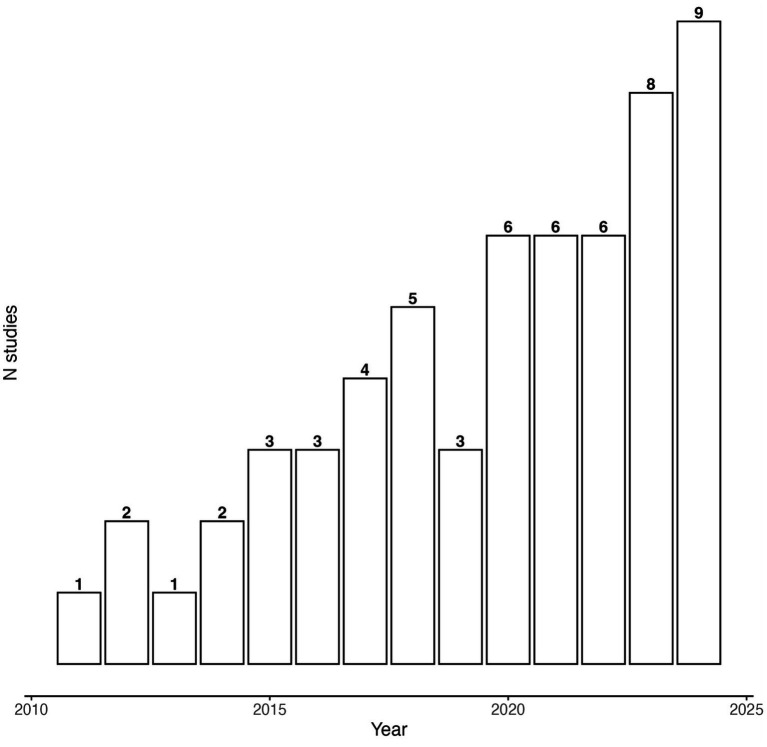
Barplot depicting number of studies per year investigating ER within forensic settings.

Table 1 provides an overview of the studies included in the review (*N* = 59). Most studies were of moderate quality (*k* = 29, 49%), 24 studies were of weak quality (*k* = 24, 41%) and six studies were of strong quality (*k* = 6, 10%). The total sample size across all studies was 13,238 (*N*_mean_ = 224, *N*_median_ = 111). Three studies reported sample sizes over *n* = 1,000 and all were conducted in large prisons in China (Gu et al., 2023; Yang et al., 2022; Zhong et al., 2022). The majority of samples consisted of only males (*k* = 34, 58%), with eight samples being composed of only females (*k* = 8, 14%) and 14 samples including both females and males (*k* = 14, 24%). Three studies did not provide information about gender distribution in their samples. The mean age across the aggregated samples was 36 years (range: 23–47). The main recruitment setting across studies was a prison (*k* = 44, 75%), followed by a forensic psychiatric hospital (*k* = 10, 17%). The remaining five studies were either conducted with mixed samples consisting of individuals from both a prison and a forensic psychiatric setting (Velotti et al., 2017; Nentjes et al., 2016), individuals on probation (Roberton et al., 2014; Roberton et al., 2015) or within a general healthcare setting for people who had a history of intimate partner violence (Nesset et al., 2021). Forensic services were mainly delivered in inpatient settings (*k* = 52, 88%). Five studies (*k* = 5, 8%) sampled forensic individuals in outpatient settings and two studies (*k* = 2, 3%) sampled individuals from both inpatient and outpatient settings (Billen et al., 2022; Velotti et al., 2017). Most studies were conducted in Italy (*k* = 10, 17%), followed by the USA (*k* = 8, 14%) and the Netherlands (*k* = 6, 10%). Figure 2 shows the total number of studies sorted by year of publication.

**Table 1:** Descriptive table of included studies.

Study	Design	Population	*N*	ER measure	QA
Bianchini et al. (2019)	CCT	Forensic psychiatry	21	DERS-36	Weak
Billen et al., 2022	Cross-sectional	Forensic psychiatry	94	DERS-16 extended	Strong
Brazão et al. (2018)	RCT	Prison population	254	ERQ	Moderate
Brazão et al. (2021)	RCT	Prison population	254	ERQ	Moderate
Brzozowski et al. (2021)	Cohort	Prison population	10	ERQ	Weak
Burghart et al. (2024)	Cross-sectional	Forensic psychiatry	50	ERQ	Moderate
Byrne et al. (2016)	CCT	Prison population	59	ERQ	Moderate
Casey et al. (2013)	Cross-sectional	Prison population	62	HR	Moderate
Choi et al. (2023)	Cross-sectional	Prison population	500	CERQ-64	Moderate
Collica-Cox et al. (2024)	Cohort	Prison population	42	ERQ	Weak
Facer-Irwin et al. (2023)	Longitudinal	Prison population	223	DERS-SF	Weak
Garofalo and Velotti (2017)	Cross-sectional	Prison population	221	DERS-36	Moderate
Garofalo and Velotti (2021)	Cross-sectional	Prison population	266	DERS-36	Moderate
Garofalo et al. (2016)	Cross-sectional	Prison population	153	DERS-36	Strong
Garofalo et al. (2020a)	Cross-sectional	Prison population	164	DERS-36	Moderate
Garofalo et al. (2020b)	Cross-sectional	Prison population	397	DERS-30	Strong
Garofalo et al. (2021)	Cross-sectional	Prison population	263	DERS-36	Moderate
Garofalo et al. (2022)	Cross-sectional	Prison population	389	DERS-36	Moderate
Gillespie et al. (2018)	Cross-sectional	Prison population	397	DERS-36	Moderate
Granados et al. (2022)	CCT	Prison population	51	TMMS-24	Weak
Grills et al. (2015)	Cohort analytic	Prison population	96	DERS-36	Weak
Gu et al. (2023)	Cross-sectional	Prison population	1,042	ERQ	Moderate
Hamilton et al. (2024)	Cross-sectional	Prison population	112	DERS-36	Weak
Hampson et al. (2024)	Cohort	Prison population	12	DERS-36	Weak
ter Harmsel et al. (2023)	Cohort	Forensic psychiatry	25	DERS-36	Weak
Hosie et al. (2022)	Cross-sectional	Prison population	129	DERS-36	Moderate
Hutchinson et al. (2017)	CCT	Prison population	57	PACS	Weak
Ifeagwazi et al. (2019)	Cross-sectional	Prison population	209	ERQ	Moderate
Ivarsson et al. (2023)	Cohort	Prison population	14	DERS-36	Moderate
Jansen (2020)	Cross-sectional	Prison population	281	ERQ	Strong
Karatzias et al. (2018)	Cross-sectional	Prison population	89	DERS-36	Weak
Khabiripooya and Namvar (2019)	Cross-sectional	Prison population	50	DERS-36	Weak
Kołodziej et al. (2022)	Cross-sectional	Prison population	152	CECS	Weak
Laporte et al. (2021)	Cross-sectional	Forensic psychiatry	98	DERS-36	Strong
McGonigal et al. (2018)	Cohort	Prison population	30	DERS-36	Weak
Meddeb et al. (2023)	Cross-sectional	Forensic psychiatry	97	DERS-36	Moderate
Messina and Calhoun (2022)	RCT	Prison population	145	DERS-36	Weak
Nentjes et al. (2016)	Cross-sectional	Mixed	84	HR, HRv, SC	Strong
Nesset et al., 2021	RCT	Healthcare	121	DERS-30	Moderate
Pato et al. (2024)	Cross-sectional	Prison population	363	CERQ-36	Moderate
Perelman et al. (2012)	Cohort analytic	Prison population	127	TMMS-48	Weak
Roberton et al. (2014)	Cross-sectional	Probation	64	DERS-36	Moderate
Roberton et al. (2015)	Cross-sectional	Probation	64	DERS-36	Moderate
Sahm et al. (2024)	Cross-sectional	Forensic psychiatry	50	ERQ + ERQ-S	Moderate
Scigala et al. (2024)	Cross-sectional	Prison population	160	DERS-36	Moderate
Shortt et al. (2014)	CCT	Prison population	47	DERS-36	Weak
Siep et al. (2019)	Cross-sectional	Forensic psychiatry	19	fMRI	Moderate
Spinelli et al. (2020)	Cohort	Forensic psychiatry	13	CERQ-36	Weak
Swain et al. (2024)	CCT	Prison population	58	DERS-36	Weak
Tonnaer et al. (2017)	Cross-sectional	Forensic psychiatry	16	fMRI	Moderate
Velotti et al. (2017)	Cross-sectional	Mixed	111	DERS-36	Moderate
Velotti et al. (2020)	Cross-sectional	Prison population	464	DERS-P	Moderate
Wahlstrom et al. (2015)	Cross-sectional	Prison population	60	DERS-36	Moderate
Walsh et al. (2011)	Cross-sectional	Prison population	160	DERS-36	Weak
Walsh et al. (2012)	Cross-sectional	Prison population	168	DERS-36	Weak
Woicik et al. (2023)	Cohort	Prison population	17	DERS-36	Weak
Yang et al. (2022)	Cross-sectional	Prison population	1,491	DERS-16	Weak
Ye et al. (2023)	Cross-sectional	Prison population	428	CECS	Weak
Zhong et al. (2022)	Cross-sectional	Prison population	2,643	ERQ	Moderate

ER, Emotion regulation; QA, Quality assessment; CCT, Controlled Clinical Trial; RCT, Randomized Controlled Trial; DERS, Difficulties in emotion regulation scale full length version; ERQ, Emotion Regulation Questionnaire; HR, heart rate; HRv, heart rate variability; SC, skin conductance; DERS-P, Difficulties in emotion regulation scale - positive; CECS, Courtauld Emotional Control Scale; CERQ, Cognitive Emotion Regulation Questionnaire; TMMS, The Trait Meta-Mood Scale; PACS, The Profile of Anger Coping Skills.

### 4.3 ER conceptualization across studies

The bulk of studies did not explicitly define ER but relied on a measurement model and, in those cases, we inferred the conceptualization based on the measure of ER that was used. Some studies, however, did to a greater extent aim to outline a definition, or a conceptualization of ER. Below follows some key conceptual differences and similarities of ER across various measurement models and individual studies.

Two ability-based frameworks of ER were identified across the review. These frameworks were operationalized through the Trait Meta-Mood scale (TMMS; Salovey et al., 1995) and the DERS framework. Two studies (Garofalo et al., 2022; Perelman et al., 2012) relied on the TMMS framework within which ER is measured along three dimensions measuring (i) attentiveness to emotions, (ii) clarity of emotions and (iii) emotion repair, or the ability to modulate emotions in the desired direction by using ER strategies. The remaining 36 studies (see Table 1) conceptualized ER along the DERS framework. While both the DERS and the TMMS conceptualize ER as a set of abilities they differ in some conceptual aspects. Firstly, the two frameworks differ in what abilities are included within the concept of ER. Secondly, whereas the DERS focuses exclusively on the dysregulation of negative emotions, the TMMS also includes items that tap the dysregulation of positive emotions within its conceptualization of ER. Within the DERS framework, although a different measure from the original DERS questionnaire, is the DERS-positive (DERS-P; Weiss et al., 2015) which was administered in one study (Velotti et al., 2020). The DERS-P shares many conceptual similarities to the original DERS questionnaire. However, contrary to the original DERS questionnaire, the DERS-P assesses the dysregulation of positive emotions (Weiss et al., 2015).

Next, to ability-based frameworks, strategy-based frameworks conceptualized ER more narrowly, focusing on one or several specific ER strategies and were measured through the Profile of Anger Coping skills (PACS; Willner et al., 2005), Courtauld Emotional Control Scale (CECS; Watson and Greer, 1983), Cognitive Emotion Regulation Questionnaire, and ERQ frameworks across the studies reviewed. All strategy-based frameworks assessed ER in relation to negative emotional states. However, the ERQ assess the use of cognitive reappraisal and suppression in relation to both positive and negative emotions. Conceptual differences between these models include the number and type of strategies that are used to capture ER as well as to which emotions are being regulated. The different strategies included across the different strategy-based frameworks are provided in Table 2. Noteworthy, type and number of ER strategy did not only vary as a function of which measurement model that was used. Studies frequently used a subset of the strategies assessed in a measurement model when investigating ER and did so for both psychometric as well as conceptual reasons.

**Table 2:** Emotion regulation strategies included within each measurement model.

ER strategy	ERQ	CERQ	CECS	PACS	Biophysiological
Suppression	X		X		X
Cognitive reappraisal	X				
Self-blame		X			
Blaming others		X			
Acceptance		X			
Refocus on planning		X			
Positive refocusing		X			
Rumination		X			
Positive reapparaisal		X			
Putting into perspective		X			
Catastrophizing		X			
Relaxation				X	
Count to 10				X	
Walk away				X	
Distraction				X	X
Ask for help				X	
Rethink the situation				X	
Use humour				X	
Be assertive				X	

Table displaying which strategies are assessed across the strategy-based measurement models. ERQ, Emotion Regulation Questionnaire; CERQ, Cognitive Emotion Regulation Questionnaire; CECS, Courtauld Emotional Control Scale; PACS, The Profile of Anger Coping skills; Biophysiological, different biophysiological indices of ER.

Whereas the ERQ measures two strategies in relation to negative and positive emotions, the CERQ measures ER along nine ER strategies in response to negative emotions. The PACS assess eight different ER strategies in response to anger and the CECS asses suppression of anger, anxiety and depressive feelings. Although some of the subscales within these measures might share the same conceptual meaning (e.g., cognitive reappraisal in the ERQ and positive reappraisal in the CERQ) the choice of ER strategy within each measurement model has been guided by different classifications of ER. Four studies, in which ER was measured through various neurobiological/physiological measures, asked participants to either employ distraction (Siep et al., 2019; Tonnaer et al., 2017) or suppression (Casey et al., 2013; Nentjes et al., 2016) and as such employed a strategy-based framework to ER. However, within these studies, ER strategies were assessed in relation to their effectiveness in reducing elicited emotions (assessed using a non-validated Likert-scale or a visual analogue scale) rather than how often ER strategies are used. In all four studies, ER was measured in relation to specific emotions, including anger, fear and happiness.

ER conceptualizations did not only differ as a function of which specific measurement model was used but also how these measurements were used and how ER was defined in each specific study. Specifically, several short-forms or adaptations of the original instruments, and the DERS in particular, were identified, impacting how ER was conceptualized. Two studies (Garofalo et al., 2019; Nesset et al., 2021) used the original DERS framework excluding items from the Awareness subscale. A second set of studies (Billen et al., 2022; Yang et al., 2022) used a short-form of the DERS in which all items tapping emotional awareness were excluded and one of these studies complemented DERS items with items from another questionnaire in their conceptualization of ER (Billen et al., 2022). One study (Roberton et al., 2015) administered the DERS-36 but only used the Awareness subscale for further analysis. While 30 studies administered the full-length DERS questionnaire, 16 studies only used the composite score and collapsed the different facets of ER in to one broadband measure of ER. The remaining 14 studies used the subscales of the DERS, and sometimes in conjunction with the composite score in their main analysis. Similarly, only a subset of the subscales from the CERQ questionnaire were used in one study (Spinelli et al., 2020). The ERQ framework was used in 11 studies but two studies (Gu et al., 2023; Zhong et al., 2022) relied only on the Cognitive Reappraisal subscale, seven studies (Brazão et al., 2021; Brazão et al., 2018; Brzozowski et al., 2021; Burghart et al., 2024; Collica-Cox et al., 2024; Ifeagwazi et al., 2019; Sahm et al., 2024) relied on both Cognitive Reappraisal and Expressive Suppression and two studies (Byrne et al., 2016; Jansen, 2020) erroneously collapsed these subscales into an overarching measure of ER.

Lastly, different ER conceptualizations made different assumptions about which ER strategies are adaptive versus maladaptive. Although the ERQ as a measure does not assume that Cognitive Reappraisal and Expressive Suppression measure an adaptive and maladaptive ER strategy respectively, this was frequently inferred across the studies reviewed. Similarly, in the two studies (Choi et al., 2023; Kołodziej et al., 2022) that relied on the CECS, both assumed Suppression to be an inherently dysfunctional ER strategy. Further, two studies that measured ER through biophysiological indices assumed Suppression to be characteristic of maladaptive ER (Nentjes et al., 2016; Siep et al., 2019). In regards to CERQ, five of the nine strategies are classified as adaptive, namely Acceptance, Positive focus, Planning, Positive reappraisal and Perspective taking, while the remaining four are conceptualized as maladaptive ER strategies: Self-blame, Rumination, Catastrophizing and Blaming others. Within the PACS framework, all eight strategies are assumed to be, more or less, functional ways of dealing with anger.

In conclusion, conceptualizations of ER can be systematically organized along four overarching dimensions. Firstly, they differ with respect to whether ER is defined as an ability or as the use of specific strategies. Secondly, they vary in terms of which emotions are regarded as the primary targets of regulation. Thirdly, they diverge in the range of strategies or abilities encompassed within the conceptualization. Lastly, they differ in how particular strategies are construed in terms of their adaptiveness.

### 4.4 ER measures across studies

ER was predominantly measured through self-report measurements (*k* = 55, 93%) with the remaining four studies (7%) employing various biophysiological measures of ER. The most commonly used self-report measure was the DERS-36 (*k* = 30, 51%), followed by the ERQ (*k* = 11, 19%). Other self-report assessments identified in the review include the CERQ, the PACS, the CECS and the TMMS. Several adaptations or short-forms were identified for a subcollection of the measurements identified. The DERS appeared in four different short-forms or adaptations. Two studies used the DERS excluding the Awareness items (Garofalo et al., 2019; Nesset et al., 2021). One study used the validated short form DERS-16 (Bjureberg et al., 2016) and a third study used the DERS-16 combined with items from UPPS-P Impulsive Behavior Scale (UPPS-P; Cyders et al., 2014) to measure ER (we will henceforth refer to this as the DERS-16 extended in the manuscript). Finally, one study administered the validated DERS-short form (DERS-SF; Kaufman et al., 2016). Besides the short-forms and adaptations described above, the DERS-P was administered in one study (Velotti et al., 2020).

The original TMMS-48 appeared in an abbreviated form with 24 items that was administered in one study (Granados et al., 2022). The three studies administering the CECS used the same version of the measurement consisting of 21 items with 7 items measuring behavioral suppression of anger, anxiety and depressive feelings, respectively. The CERQ was administered in three studies with two studies administering the original 36-item based questionnaire and one administering a prolonged version of the CERQ with 64 items. The original CERQ questionnaire measures nine cognitive ER strategies through four items each. The PACS was administered in only one study and consisted of eight ER strategies that an individual could employ when experiencing anger. One short-version of the ERQ was identified (Sahm et al., 2024) where the ERQ was reduced to six items instead of the original 10. Four studies examined ER through various forms of biophysiological measures (*k* = 4, 8%) including: fMRI (*k* = 2); heart rate (HR; *k* = 2); heart rate variability (HRv, *k* = 1) and skin conductance (SC, *k* = 1). The two studies that employed fMRI techniques (Siep et al., 2019; Tonnaer et al., 2017) both used an adaptation of the Anger Articulated Thoughts during Simulated Situations paradigm (ATSS; Davison et al., 1995) in which participants listened to audiotapes of stories with varying affective content and were instructed to distract themselves (i.e., down-regulate) during the emotion regulation phase of the paradigm. The remaining two studies (Casey et al., 2013; Nentjes et al., 2016) used visual stimuli (e.g., film and/or pictures) to elicit emotions and provided participants with instructions to either use suppression as a strategy to down-regulate their emotional response or to try to maintain their emotional response (i.e., no modulation of affect). Different physiological measures of ER were employed in the last two studies with one focusing solely on heart rate (Casey et al., 2013), whereas the other study measured heart rate along with heart rate variability and skin conductance (Nentjes et al., 2016).

Three studies administered multiple measures of ER (Gillespie et al., 2018; Sahm et al., 2024; Velotti et al., 2020). In all three studies, two self-report measurements were administered to the same sample. In the study by Gillespie et al. (2018) both the ERQ and the DERS-36 were administered. In the study by Velotti et al. (2020) the DERS-P and the original DERS questionnaire were administered. In the study by Sahm et al. (2024) two versions of the ERQ were administered in which the ERQ-Short from (ERQ-S) was psychometrically evaluated and compared to the full length ERQ.

**Figure 3:**
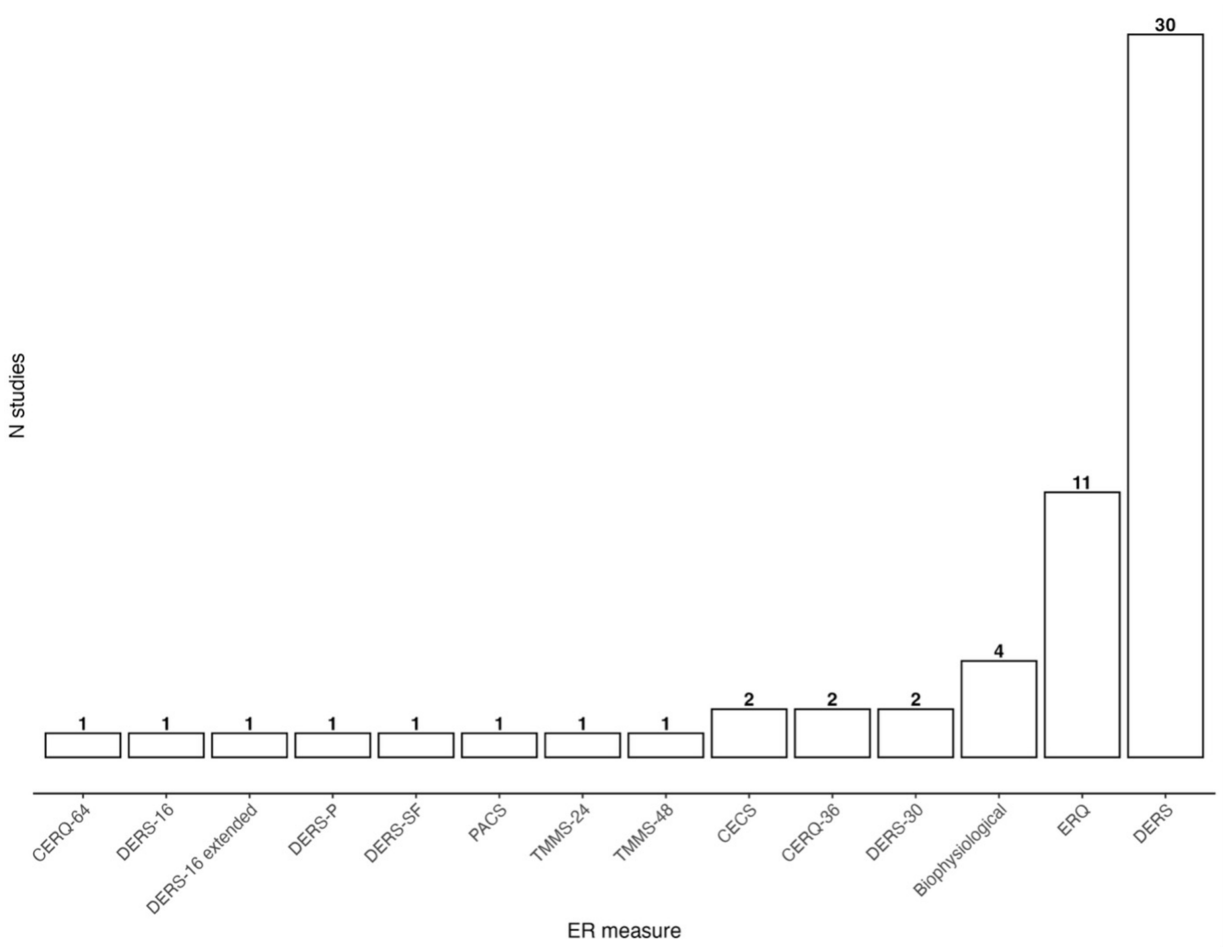
Barplot showing the frequency of each measurement model across studies. CERQ, Cognitive Emotion Regulation Questionnaire; DERS-16, Difficulties in emotion regulation scale 16 items; DERS-16 extended, DERS 16 and additional items from the UPPS-P Impulsive Behavior Scale; DERS-P, DERS-Positive; DERS-SF, DERS-short form; PACS, The Profile of Anger Coping Skills; TMMS, The Trait Meta-Mood Scale; CECS, Courtauld Emotional Control Scale; CERQ, Cognitive Emotion Regulation Questionnaire; DERS-30, excluding items from the Awareness subscale; Biophysiological, includes various physiological measures of ER; ERQ, Emotion Regulation Questionnaire; DERS, Difficluties in emotion regulation scale with 36 items.

In summary, seven different measurement models of ER were identified across the studies included in this review. The DERS framework and its various declensions was by far and large the most common measure of ER, followed by the ERQ. Taken together, the DERS and the ERQ frameworks account for almost 80% of all measures of ER. For a visual display over ER measures used across the identified studies see Figure 3.

### 4.5 Reliability of ER measures

**Figure 4:**
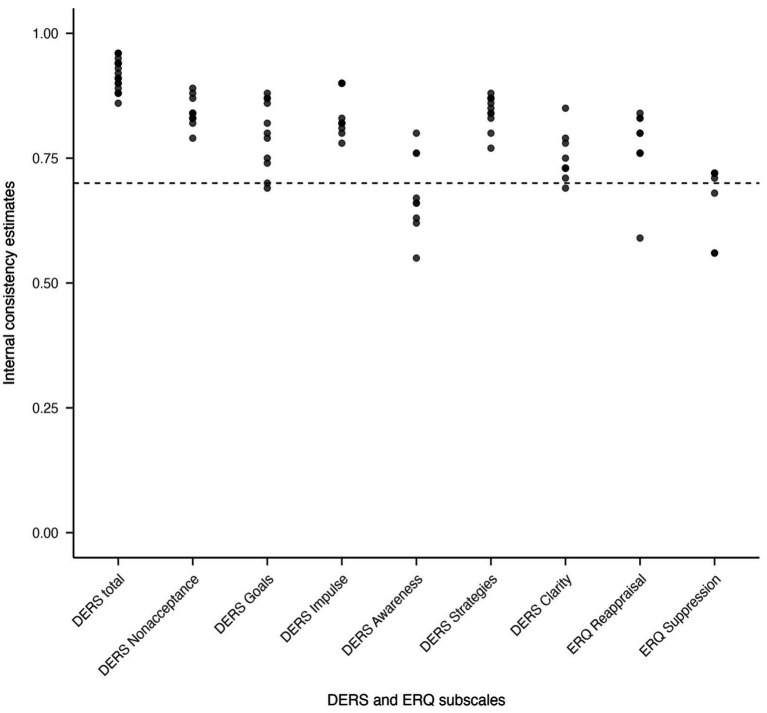
Scatterplot depicting internal consistency for DERS-36 and ERQ subscales.

In total 38 (64%) studies reported internal consistency of ER measures for their samples. Cronbach’s alpha was the predominant estimate of internal consistency as this was reported in 37 studies. Only one study reported McDonalds’ omega (Ye et al., 2023). Internal consistency was not estimated in those studies that conceptualized ER through the PACS, TMMS-24, DERS-SF or the DERS-16 extended. In the case of the DERS-16 extended, internal consistency was reported for 11 items after having performed a factor analysis but no report of internal consistency for the actual measure as it was used in the analysis was reported. Nor were any estimates of reliability reported in the studies that employed biophysiological measures of ER. In the two studies that administered the CERQ-36, exact estimates of internal consistency could not be extracted due to how data were reported. The CERQ-64 was reported to have good internal consistency at the composite level (*α*: 0.87) with a range of 0.59–0.80 at the subscale level. Internal consistency for the CECS was reported in two studies with internal consistency for the composite score estimated at α = 0.87 in one study and *ω* = 0.88 in the second study. One study also reported internal consistency for individual subscales of the CECS (α range; 0.77–0.80). Internal consistency for the TMMS-48 was only reported for individual subscales (α_attention_ = 0.86, α_clarity_ = 0.86, α_repair_ = 0.86). Internal consistency for the DERS-P was reported for both the composite score and subscale scores (α_composite_ = 0.90, α_nonacceptance_ = 0.80, α_goals_ = 0.80, α_impulse_ = 0.86). Similarly, internal consistency for the DERS-16 was reported at both composite and subscale scores (α_composite_ = 0.94, α_nonacceptance_ = 0.88, α_goals_ = 0.86, α_impulse_ = 0.90, α_strategies_ = 0.80, α_clarity_ = 0.85). Internal consistency for the ERQ-S was reported for the two subscales (α_reappraisal_ = 0.63, α_suppression_ = 0.65). The two studies that used the DERS framework excluding items from the Awareness subscale justified doing so partly through the low internal consistency of the subscale. One of the studies (Nesset et al., 2021) relying on the DERS-30 reported internal consistency for the composite and subscale scores as α > 0.90. The second study (Garofalo et al., 2019) reported precise estimates of internal consistency (α_composite_ = 0.91, α_nonacceptance_ = 0.82, α_goals_ = 0.74, α_impulse_ = 0.80, α_strategies_ = 0.84, α_clarity_ = 0.73). Amongst the 29 studies that relied on the DERS-36 and the 11 studies that relied on the ERQ-10, 19 and 8 studies, respectively, reported estimates on internal consistency. These estimates are visually displayed in Figure 4.

### 4.6 Validity of ER measures

**Figure 5:**
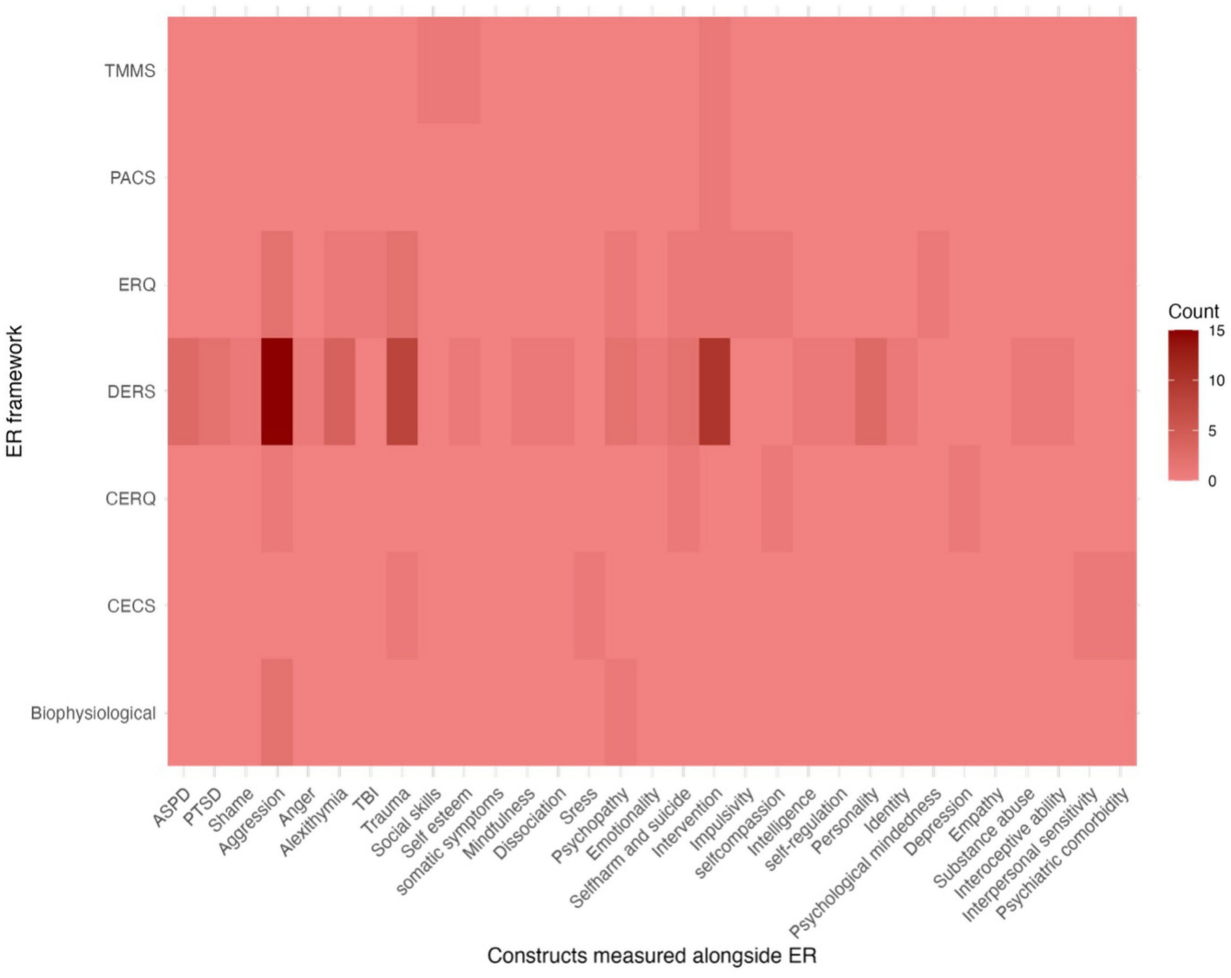
Heatmap showing which constructs and how often they are measured in conjunction with emotion regulation frameworks. TMMS, The Trait Meta-Mood Scale; PACS, The Profile of Anger Coping Skills; ERQ, Emotion Regulation Questionnaire; DERS, Difficluties in emotion regulation scale; CERQ, Cognitive Emotion Regulation Questionnaire; CECS, Courtauld Emotional Control Scale; Biophysiological, includes various physiological measures of ER; TBI, traumatic brain injury.

Out of the 59 studies included in the review, 24 individual studies provided correlation coefficients between ER and other constructs alongside which ER was measured. One study did not provide any support of validity, 19 studies provided mixed support of validity and four studies provided support of validity. Specifically, ER was measured alongside 31 different constructs across the 59 studies. However, the majority of these constructs were only measured once in relation to ER (*k* = 19, 61%). The most common constructs alongside which ER was measured were aggression (*k* = 20), followed by an intervention (*k* = 17) and trauma (*k* = 11) (see Figure 5).

## 5 Discussion

Research on ER within forensic psychological research has been growing over the past two decades. Compared to similar reviews conducted within children and adolescent samples wherein research into ER can be traced back to the 1980’s (Adrian et al., 2011), no records were found through our search prior to 2010. This attests to the relatively recent surge in ER research within forensic settings compared to other related fields within psychological research. This review of how ER is conceptualized and measured within forensic settings provides valuable insight in to how ER research may further develop in these settings, providing an integrated conceptualization of ER, highlighting potential areas of improvement and what challenges lay ahead.

As evidenced through this review, different conceptualizations of ER are adopted within the field of forensic psychology. While an argument could be made that this heterogeneity hampers research to move forward (e.g., Elson et al., 2023), different conceptualizations and measurement models could be viewed from a more integrative lens where ER abilities may be considered a higher order process of ER strategies (Tull and Aldao, 2015). For instance, it is likely that the continuous use of suppression may influence ER abilities such as emotional awareness and that, similarly, a greater degree of emotional awareness may help an individual to resolve emotional issues and be less inclined to employ an ER strategy such as suppression. In other words, ER strategies and ER abilities are not isolated processes, albeit distinguishable from one another. However, we noted that also within these two broad approaches to ER, differences exist as to what abilities and which strategies are considered to constitute ER, although these differences do not appear to substantially alter the fundamental meaning attributed to ER, but rather define the scope (broader or narrower) that each study has selected to embrace. Moreover, different ER measures implied different emotions to be regulated. Although ER findings may not vary drastically between emotions, these conceptual differences between measurement models highlight the importance of studies to clearly define their intended conceptualization of ER explicitly. This finding echoes findings from other systematic reviews on ER that have found that the underlying model of ER is rarely explicitly defined (Martínez-Priego et al., 2024). Moving forward, and given the variety of definitions of ER that exist, we recommend that studies include a clear conceptual definition of the construct guiding their research aim. This could facilitate integration across studies as well as examination of within-study consistency between theoretical conceptualizations and methods of operationalization.

Considering the challenges in recruiting participants within forensic psychological research (Pedersen et al., 2021) and the advantages in using more economic questionnaires to reduce participant burden, it was surprising that no more than three studies used one of the many DERS short-forms available. This could be due to the relative recency of short-forms development, or to the limitations of capturing subscale scores with a smaller set of items. It is worth noting that, although many studies used the same measures, their use varied across studies. While the DERS can be defensibly used both at the total score and subscale level, depending on the study aims and hypotheses, it was surprising to note that two studies included in our review employed the total ERQ score in their analysis (Byrne et al., 2016; Jansen, 2020). Considering that the ERQ measures two different emotion regulation strategies that are considered theoretically different in terms of adaptability, functioning and well-being (Gross and John, 2003), using the sum score for the ERQ in studies undermines the potential theoretical contribution to the understanding of emotion regulation within the forensic context.

The bulk of knowledge on ER within forensic settings relies heavily on the unimodal measure of self-report questionnaires. The reliance on self-report measures were addressed in several discussion sections across the studies reviewed (e.g., Bianchini et al., 2019; Velotti et al., 2017) and broadening how ER is assessed within studies—prioritizing multi-method assessment of ER—may be an important avenue of further development. In the studies that examined ER using biophysiological indices, researchers sought to standardize the emotional cue by employing experimental paradigms and instructed participants to engage in prespecified ER strategies. Although such procedures may reliably induce the target emotion in participants, the induced emotion may not be identical to that experienced in a real-world setting (Campos et al., 2011). Moreover, such controlled settings, with standardized instructions of what ER strategy to employ, may say very little about how ER strategies are used in every-day life. One line of research worthy of further study would be studies that aim to capture and sample ER dynamics through ecological momentary assessment (EMA) methods. Although, the use of such methods increasingly relies on the use of smart phones, which are not usually accessible to people in prisons or in forensic psychiatric hospitals. However, EMA studies have previously been conducted with both forensic psychiatric outpatients (ter Harmsel et al., 2023) and inpatients (Habets et al., 2022) although sample size tends to fall on the small side, and participants are likely a selected and non-representative sub-group. Finally, it is worth mentioning that no studies included measures of ER based on interviews or clinical-ratings, although these are generally rarely used in ER research, they may prove especially useful in samples characterized by limited insight like those drawn from forensic populations.

While many studies did include sample-specific reliability estimates, the reporting rate was not as consistent as would be expected, given that such information is considered standard in scientific publishing. Further, in our review we did not retrieve any formal validation studies that have attempted to corroborate the measurement models in forensic settings specifically, typically taking for granted that measures developed in community or clinical populations would translate to forensic samples. The questionnaires that had been developed within the DERS framework exhibited conventionally good estimates of internal consistency. However, the Awareness subscale was found to exhibit lower levels of internal consistency, as has been previously noted in the literature (Bardeen et al., 2012; Hallion et al., 2018; McVey et al., 2022; Osborne et al., 2017; Raimondi et al., 2024). This has partly motivated some studies to exclude the Awareness subscale from the DERS and there is ongoing debate as to whether the Awareness subscale is to be considered part of the higher-order construct of emotion dysregulation as measured through the DERS (e.g., Bardeen et al., 2012). Internal consistency estimates for the ERQ subscales were somewhat lower than those reported for measures within the DERS framework. One possible explanation is that the ERQ items capture emotion regulation across both positive and negative emotional contexts, which may involve partly distinct processes and therefore reduce item covariance. An alternative explanation may be that the use of ER strategies may not be uniform across situations. In other words, if ER strategies are context dependent, they may lack internal consistency for a reason. For the remaining self-report measures identified in this review, reliability estimates were either not reported (TMMS-24, PACS, DERS-16 Extended, DERS-SF) or based on too few studies (CERQ-64, CERQ-36, TMMS-48, CECS) to draw any firm conclusions.

Moreover, we noted that in some cases internal consistency was reported for the composite score when analysis were performed at the subscale level or vice versa. None of the studies that employed a biophysiological measure of ER reported measures of reliability. The lack of reliability reporting for laboratory measures, broadly speaking, has previously been criticized in the literature (Lilienfeld and Strother, 2020). It is reasonable to assume that due to the situational uniqueness of laboratory measures that there may be a host of transient situational factors that play in when assessing emotion regulation. Preferably, authors would consistently report estimates of internal consistency for their specific study and at the measurement level most fit for their study.

Our review seems to suggest that ER measures used in forensic settings, even though not formally validated in terms of their factor structure and even though flawed by an inconsistent reporting of their internal consistency, do show evidence of construct validity. Specifically, we found that the bulk of studies provided some degree of support for the validity of ER measures, showing patterns of associations with indices of psychopathology or maladaptive functioning—widely defined—in line with conceptual expectations. It should be noted that in those studies that employed a biophysiological assessment of ER no correlations between ER and other constructs of interest were provided why validity could not be appraised in these studies. Considering the breadth of constructs examined in conjunction with ER (see Figure 5), synthesizing our results across studies is not possible in this review and would require a different study design. However, our integrative approach provides promising evidence that ER is a putative important transdiagnostic feature across the broad spectrum of mental health problems. Considering that forensic samples suffer from high levels of psychiatric comorbidity (Laporte et al., 2021) coupled with aggressive and antisocial behavior, indices of functioning that revolve around these aspects were especially prevalent. Meta-analytical studies are accumulating that corroborate the association between ER and other constructs of clinical relevance within forensic psychological research such as psychopathy (Velotti et al., 2024) recidivism (Seto et al., 2023), substance abuse (Stellern et al., 2023) and intimate partner violence (Maloney et al., 2023; Neilson et al., 2023). Considering that aggression and trauma were the most common constructs alongside which ER was measured, it may be feasible for future research to meta-analytically estimate the association between ER and aggression and ER and trauma. Finally, it was notable that, with few exceptions, ER was investigated in relation to maladaptive or psychopathological functioning. Even in studies that represented an exception by focusing on associations that ER had with self-esteem or intelligence, these were framed in the direction of impairment (i.e., low self-esteem, low intelligence). Research on ER in forensic settings would likely benefit from an increased focus on how *better* ER is associated with *adaptive* functioning, in an attempt to stress how ER is relevant not only for reducing problems and dysfunctions, but also for promoting well-being.

### 5.1 Limitations

The findings from this review should be read with consideration of its limitations. Firstly, the generalizability of our findings should be read in relation to how we defined ER in the protocol. We used an approach similar to other systematic reviews and meta-analysis of emotion regulation (e.g., Maloney et al., 2023; Sloan et al., 2017). Similarly, it is possible that we did not include studies that may be relevant for this review but that did not discuss their aims or their findings in relation to ER specifically. However, we wanted to prioritize specificity of the studies included over their quantity. In addition, had we included any study that loosely refer to ER, we might have underestimated the quality of ER studies in forensic settings, because it is understandable that if ER was only a secondary or cursory topic, less attention would be paid to its conceptualization and measurement. Secondly, our definition of a forensic setting includes admittedly a large variety of individuals that differ in terms of their mental health profiles which consequently introduces a large degree of heterogeneity. However, to our knowledge, there is no standard demarcation of what constitutes a forensic setting or population across countries and jurisdictions. While acknowledging that other researchers may have relied on a different definition for what constitutes a forensic setting we have aimed to be consistent in which studies that were selected within this systematic review.

Thirdly, validity was hard to gauge in relation to specific measures. Due to this, we focused our attention at the larger picture, examining correlation coefficients between ER and other relevant constructs alongside which ER was measured based on conceptual expectations. Aggregating across different operationalizations as was done when summarizing the evidence for validity, as well as coding studies as either supporting, not supporting or providing mixed support of construct validity may overlook nuances in the data. Moreover, only one author coded the data used to appraise validity which may introduce bias in the data-extraction procedure.

## 6 Conclusion

ER is increasingly being studied within applied forensic settings and has been measured alongside a wealth of different constructs. The field seems to be converging on the use of the DERS and ERQ frameworks when measuring ER in forensic settings. These two frameworks represent two broad conceptual approaches to ER, one focused on ER as a trait-based disposition to successfully or unsuccessfully regulate emotions, and one focused on the use of specific ER strategies. Nonetheless, this review shows that measurement models and studies differ in terms of what strategies and what abilities are included within the concept of ER as well as which emotions are considered to be regulated within each framework. Reliability was too infrequently reported, but when it was reported, it was found to be within acceptable ranges by conventional cut-offs (e.g., Nunnally, 1978). However, lower reliability estimates were reported specifically for the Suppression subscale in the ERQ and the Awareness subscale in the DERS-36, and questions remain as to whether the over 30% of studies that did not include reliability estimates were failing to report low estimates. Most studies were found to provide either conclusive or mixed support of construct validity for ER which aligns well with previous research that has framed ER as a transdiagnostic factor across a wide spectrum of psychopathology. In conclusion, this study highlights the varied conceptualization and measurement of ER across forensic settings. Based on our findings, we recommend that studies consistently report the reliability of their ER measures and explicitly define their conceptualization of ER. These steps may improve the interpretation of how conceptualizations align with measurement models and facilitate a more critical evaluation of ER findings.

## Data Availability

The original contributions presented in the study are included in the article/supplementary material, further inquiries can be directed to the corresponding author.

## Footnotes

1
https://osf.io/q65ga/?view_only=076cf5c816d740a0ad23fb7e1ac041b4
